# Attention‐deficit/hyperactivity disorder and white matter microstructure: The importance of dimensional analyses and sex differences

**DOI:** 10.1002/jcv2.12109

**Published:** 2022-11-15

**Authors:** Scott A. Jones, Bonnie J. Nagel, Joel T. Nigg, Sarah L. Karalunas

**Affiliations:** ^1^ Department of Psychiatry Oregon Health & Science University Portland Oregon USA; ^2^ Department of Behavioral Neuroscience Oregon Health & Science University Portland Oregon USA; ^3^ Department of Psychological Sciences Purdue University West Lafayette Indiana USA

**Keywords:** ADHD, brain development, brain imaging, sex differences

## Abstract

**Background:**

Attention‐deficit/hyperactive disorder (ADHD) has substantial heterogeneity in clinical presentation. A potentially important clue may be variation in brain microstructure. Using fractional anisotropy (FA), previous studies have produced equivocal results in relation to ADHD. This may be due to insufficient consideration of possible sex differences and ADHD's multi‐componential nature.

**Methods:**

Using whole‐brain analyses, we investigated the association between FA and both ADHD diagnosis and ADHD symptom domains in a well‐characterized, ADHD (*n* = 234; 32% female youth) and non‐ADHD (*n* = 177; 52% female youth), case‐control cohort (ages 7–12). Sex‐specific effects were tested.

**Results:**

No ADHD group differences were found using categorical assessment of ADHD without consideration of moderators. However, dimensional analyses found total symptoms were associated with higher FA in the superior corona radiata. Further, *inattention* symptoms were associated with higher FA in the corpus callosum and ansa lenticularis, and lower FA in the superior longitudinal fasciculus (SLF), after controlling for overlap with hyperactivity‐impulsivity. *Hyperactivity‐impulsivity* symptoms were associated with higher FA in the SLF, and lower FA in the superior cerebellar peduncles, after control for overlap with inattention. Meanwhile, both categorical and dimensional analyses revealed ADHD‐by‐sex interactions (voxel‐wise *p* < .01). Girls with ADHD had higher FA, but boys with ADHD had lower FA (or no effect), compared to their same‐sex peers, in the bilateral anterior corona radiata. Further, higher ADHD symptom severity was associated with higher FA in girls, but lower FA in boys, in the anterior and posterior corona radiata and cerebral peduncles.

**Conclusions:**

ADHD symptom domains appear to be differentially related to white matter microstructure, highlighting the multi‐componential nature of the disorder. Further, sex differences will be crucial to consider in future studies characterizing ADHD‐related differences in white matter microstructure.

## Graphical abstract

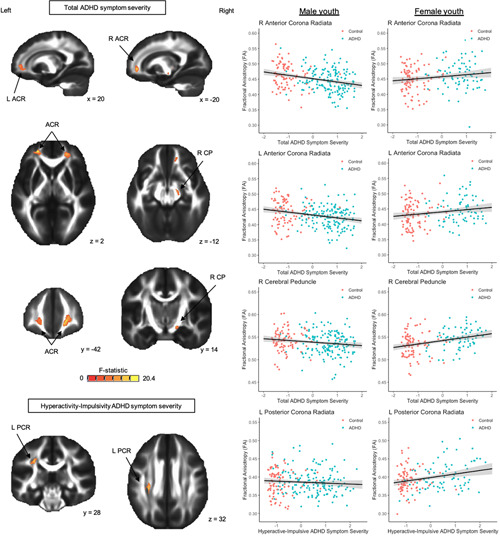

Sex differences in ADHD have not been adequately considered, particularly for studies of fractional anisotropy (FA), a measure of white matter microstructural development. Results here demonstrate that ADHD symptom domains are differentially related to FA, highlighting the multi‐componential nature of the disorder. Males and females showed opposing patterns of FA, and studies of ADHD and white matter microstructure should consider symptom heterogeneity and sex to develop neurobiologically plausible models of ADHD brain development.

**Key points**

- Previous investigations into the relationship between attention‐deficit/hyperactive disorder (ADHD) and white matter microstructure have produced equivocal findings due to possible sex‐specific effects and ADHD's multi‐componential nature.
- Exploratory whole‐brain analyses in one of the largest pre‐adolescent case‐control cohorts to‐date confirmed sex‐specific effects of ADHD on white matter microstructure, particularly in the prefrontal cortex. Boys with ADHD showed consistently lower fractional anisotropy (FA) and girls with ADHD showed consistently higher FA than their typically‐developing peers.
- Dimensional analyses of separate symptom domains reveal unique, and sometimes opposing, associations in white matter microstructure.
- Future studies of white matter microstructure in ADHD are encouraged to include examinations of symptom domains and sex differences.

## INTRODUCTION

Attention‐deficit/hyperactive disorder (ADHD) is one of the most common and costly neurodevelopmental psychiatric disorders (American Psychiatric Association, 2013). Despite remarkable growth in our understanding of neurobiology, the fundamental pathophysiology of ADHD is still not fully understood, partly due to heterogeneity in this population, both in core symptoms and associated features.

One important, clinically‐relevant microstructural neurobiological marker in ADHD is fractional anisotropy (FA), estimated using diffusion tensor imaging (DTI). The most common white matter microstructural metric, FA has historically been thought to reflect microstructural components of white matter, such as fiber density, axonal diameter, and myelination (Hagmann et al., 2006). However, while associations between FA and axonal density have been recently confirmed (Friedrich et al., 2020), FA may also be related to glial cells swelling and neuronal excitotoxicity (Lee et al., 2021). Despite limitations in the interpretability, FA is an often‐utilized metric that may provide useful in understanding ADHD neurobiology and potentially lead to treatment targets (Bouziane et al., 2019).

Previous DTI studies have produced equivocal results about the directionality of ADHD‐related effects in youth. An early meta‐analysis found differences in FA between youth with and without ADHD in the anterior corona radiata (ACR), internal capsule, forceps minor and cerebellum, regions that may subserve fronto‐striatal‐cerebellar neurocircuitry and relate to cognitive and motor functions in ADHD (van Ewijk et al., 2012). However, the contributing studies reported both higher and lower FA in ADHD in their identified regions, and subsequent exploratory studies have continued to reveal disparate findings (Ameis et al., 2016; Chen, Huang et al., 2015; Chuang et al., 2013; King et al., 2015; van Ewijk et al., 2014; Wu et al., 2017).

Several factors may explain mixed findings in ADHD. First, symptom presentations are heterogeneous; children may differ in the predominant symptoms (inattention, hyperactivity‐impulsivity, or both) and in severity of symptoms (diagnostic criteria are met with 6–18 symptoms in childhood). Studies using total ADHD symptom severity composites have also identified both higher (e.g., van Ewijk et al., 2014) and lower FA (e.g., Albaugh et al., 2019). However, inattention and hyperactivity‐impulsivity symptoms, while correlated (e.g., Goh et al., 2020), are partially dissociable, particularly with regard to external correlates (e.g., Martel et al., 2011). Preliminary studies suggest that hyperactivity‐impulsivity is associated with *higher* FA (King et al., 2015; Wu et al., 2017), while inattention is associated with *lower* FA (Ashtari et al., 2005; Witt & Stevens, 2015), though in likely non‐overlapping brain regions, a finding that warrants further investigation.

Second, sex‐specific differences in FA may exist. Despite notable overlap in correlates (Loyer Carbonneau et al., 2021), there are sex differences in ADHD symptom presentation, severity, and developmental course (Murray et al., 2019). Further, there are sex differences in typical white matter development (Kaczkurkin et al., 2019), and sex‐specific effects of ADHD diagnosis have been reported for gray matter morphometry (e.g., Seymour et al., 2017) and functional connectivity (e.g., Rosch et al., 2018). Preliminary of white matter microstructure suggest male youth with ADHD had *lower* FA, whereas female youth with ADHD had *higher* FA, compared to controls, in regions such as the corticospinal tract, inferior fronto‐occipital, superior and inferior longitudinal, and uncinate fasciculi, motor cortex, and orbitofrontal cortex (Jacobson et al., 2015; King et al., 2015). However, most previous studies lacked power to investigate sex differences, or investigated ADHD‐related effects only in boys, perhaps because ADHD is more common in male youth (Cuffe et al., 2005). The present study sought to expand these preliminary reports in a larger sample with a greater number of female youth.

Given these limitations, the current study sought to address three questions in the largest case‐control sample to‐date of pre‐adolescent children with diffusion imaging data. Hypotheses included: (1) categorical ADHD diagnosis will be associated with widespread *lower* FA, like commonly found in smaller recent samples (Ameis et al., 2016; Chuang et al., 2013; King et al., 2015). (2) dimensional total ADHD symptom severity will be associated with both higher and lower FA in regions not detectable in case‐control comparison. Further, based on prior literature (Ashtari et al., 2005; King et al., 2015; Witt & Stevens, 2015; Wu et al., 2017), we expected inattention and hyperactivity‐impulsivity symptoms (when controlling for the other symptom domain) to be differentially associated with FA in a region‐specific manner. (3) FA will be *lower* in boys with ADHD and *higher* in girls with ADHD compared to same‐sex typically‐developing children, specifically in inferior frontal regions and other major white matter tracts found previously (i.e., corticospinal tract, superior longitudinal fasciculus [SLF]) (Jacobson et al., 2015; King et al., 2015).

## METHODS

### Recruitment and diagnostic assignment

Children (*n* = 618) ages 7–12 were recruited from the local community. After screening for sufficient data, motion contamination during imaging, and restriction to tight criteria for ADHD and non‐ADHD cases (see Supporting Information S1), the final sample included 411 participants (234 with ADHD; 177 without ADHD). DTI in a small subset of this sample (*n* = 36) was reported previously (Nagel et al., 2011).

Exclusionary criteria included: left‐handedness, history of seizure, head injury with loss of conscious (or other major medical conditions), substance use disorder, prior diagnosis of autism spectrum disorder or psychosis, current use of non‐stimulant psychoactive medications, estimated IQ < 70, or MRI contraindications. Participants prescribed stimulant medication were asked to wash‐out for at least five medication half‐lives (24–48 h), prior to scanning.

ADHD diagnoses were established in a multi‐step process using standardized, nationally‐normed rating scales from parents and teachers, parent semi‐structured clinical interviews, child intellectual testing, and clinical observations (see Supporting Information S1). Final ADHD diagnoses and all other comorbid disorders were made independently by a diagnostic team (board certified psychiatrist and licensed clinical psychologist). Diagnostic assignments achieved acceptable agreement for ADHD diagnosis (kappa > 0.88) and all other disorders with >5% base rate in the sample (kappa > 0.70).

### ADHD symptom severity

Multi‐indicator latent variable scores for ADHD symptom severity were computed as described previously (Nigg et al., 2020). An overall ADHD symptom severity latent variable, and two separate latent variables for inattention and hyperactivity‐impulsivity, were estimated using the parent‐reported ADHD measures (see Supporting Information S1).

### Image acquisition

Participants were scanned on a 3T Siemens Magnetom Tim Trio with a 12‐channel head coil. Diffusion‐weighted imaging (DWI) scans were collected using a whole‐brain, high‐angular resolution, echo‐planer imaging (EPI) sequence (repetition time = 9100 ms, echo time = 88 ms, field of view = 256 mm^2^, slices = 72, slice thickness = 2 mm) with gradient‐encoding pulses in 30 directions (b‐value = 1000 s/mm^2^) and six images collected with a b‐value of 0 s/mm^2^. Participants received either two (*n* = 70, scan time = 11:24) or three (*n* = 341, scan time = 16:52) DWI runs. A diffusion field map was also acquired (repetition time = 790 ms, echo time 1 = 5.19 ms, echo time 2 = 7.56 ms, flip angle = 60°, field of view = 240 mm^2^, slices = 72, slice thickness = 2 mm, scan time = 3:13).

### Image quality assessment

All DWI runs underwent strict visual inspection, as described previously (Jones et al., 2019; Roalf et al., 2016). As failure to exclude data with motion‐related artifacts can negatively bias FA, demonstrated here (Figure S1) and by others (Roalf et al., 2016), affected DWI volumes were excluded. However, because complete exclusion of diffusion directions in DWI can positively bias FA (Chen, Tymofiyeva et al., 2015), participants were excluded completely if >20% of volumes (six directions) contained motion artifacts in all DWI runs within a subject (see Supporting Information S1). Youth with ADHD excluded from DTI analyses did not differ in ADHD symptom severity from those included in this study (see Supporting Information S1). This motion scrubbing procedure resulted in the inclusion of fewer remaining diffusion volumes, and a lower average signal‐to‐noise ratio in the cleaned data for those with ADHD, compared to those without ADHD (see Supporting Information S1). Sensitivity analyses considered the effects of signal‐to‐noise ratio, and the number of useable diffusion volumes (see below).

### Image processing

Image processing was carried out via the FMRIB Software Library Diffusion Toolbox (v 5.0.11) practices, as described previously (Jones et al., 2019). Briefly, all DWI runs within a scan were concatenated, corrected for EPI and eddy current‐induced field distortions, intensity inhomogeneities, and head motion (Andersson & Sotiropoulos, 2016), prior to estimating the eigenvalues of the diffusion tensor, and generating FA maps for each subject (Smith et al., 2004). Advanced Normalization Tools (ANTs) algorithms were used to register FA maps using a single‐step interpolation that included registration to an unbiased study‐specific template (see Supporting Information S1) and non‐linear transformation to standard Montreal Neurological Institute space (Avants et al., 2011). A Gaussian blur (sigma = 1 mm) was applied to all FA maps, and a white matter mask (mean FA > 0.3, across the entire sample) was created prior to voxel‐wise analyses, to restrict analyses to primarily white matter (Figure S2).

### Image analyses

Voxel‐wise analyses were carried out using the Analysis of Functional Neuroimages' 3dMVM to examine categorical (Hypothesis 1) and dimensional (Hypothesis 2) effects of ADHD on FA, as well as differences by sex (Hypothesis 3), controlling for age. Though inattention and hyperactivity‐impulsivity were highly correlated (*r* = 0.77, *p* < .001), both symptom domains were modeled simultaneously in dimensional analyses to identify regions unique to each domain. Main effects and interactions from each model were identified voxel‐wise (*p* < .01) and FWE‐corrected (alpha < 0.05; minimum cluster size = 298 voxels) (Cox et al., 2017). Voxel‐wise models included:

(1) FA ∼ diagnosis + sex + age
(2) FA ∼ diagnosis + sex + diagnosis × sex + age
(3) FA ∼ total ADHD symptoms + sex + age
(4) FA ∼ total ADHD symptoms + sex + total ADHD symptoms × sex + age
(5) FA ∼ inattention symptoms + hyperactivity‐impulsivity symptoms + sex + age
(6) FA ∼ inattention symptoms + hyperactivity‐impulsivity symptoms + sex + inattention symptoms × sex + hyperactivity‐impulsivity symptoms × sex + age

Sex‐by‐ADHD interactions were interpreted from Models 2, 4, and 6, while overall main effects of ADHD status or symptom severity were interpreted from Models 1, 3 and 5, as were significant main effects of sex (Table S1 and Figure S3). ADHD effects found without modeling sex of child can also be found in the Supporting Information S1 (Table S2).

Following voxel‐wise analyses, mean FA for each significant cluster was calculated, to interpret interaction terms, visualize results, and check for outliers. In regions where outliers were present, voxel‐wise models were rerun with those subjects excluded (see Supporting Information S1).

## RESULTS

### Sample characteristics

Clinical and demographic variables are provided in Table 1. The group with ADHD was slightly older and had a higher proportion of boys, but total (*p* = .98), inattention (*p* = .96) and hyperactivity‐impulsivity (*p* = .98) ADHD symptoms severity scores did not differ by sex, after controlling for ADHD status. The group with ADHD also had more associated psychiatric conditions and lower estimated IQ. IQ was not included as a covariate in DTI analyses because covarying features that reflect population‐level differences is not generally appropriate (Dennis et al., 2009). There were 48 sibling pairs (and one triad) included in this sample. Sensitivity analyses considered the effects of comorbidity, medication status, and familial relatedness (see below).

**TABLE 1:** Sample characterization

	Control	Attention‐deficit/hyperactive disorder (ADHD)	Statistic	*p* value
*N*	177	234		
Age: Mean (SD)	10.0 (1.6)	10.3 (1.5)	*t*(409) = 2.07	.038
Sex: *N* (%)				
Male	85 (48%)	158 (68%)		
Female	92 (52%)	76 (32%)	*x*2(1) = 15.06	<.001
Estimated full scale IQ: Mean (SD)	115.6 (12.9)	108.1 (13.9)	*t*(408) = 5.59	<.001
Word reading: Mean (SD)	114.5 (10.6)	105.7 (12.7)	*t*(409) = 7.43	<.001
Household income: Median (thousands)	$75–$100	$75–$100	*x*2(1) = 2.72	.099
Hispanic/Latino: *N* (%)	10 (6%)	15 (6%)	*x*2(1) = 0.01	.912
Race: *N* (%)				
American Indian/Alaska Native	0 (0%)	1 (<1%)		
Asian/East Indian	3 (2%)	6 (3%)		
Black	0 (0%)	6 (3%)		
White/Middle Eastern	154 (87%)	199 (85%)		
More than one race	20 (11%)	21(9%)		
Unknown/Unreported	0 (0%)	1 (<1%)	*x*2(4) = 6.23	.183
ADHD symptom severity: Mean (SD)				
Inattentive	−1.06 (0.41)	0.65 (0.62)	*t*(409) = 31.91	<.001
Hyperactive‐impulsive	−0.89 (0.43)	0.50 (0.81)	*t*(409) = 20.85	<.001
Combined	−1.13 (0.43)	0.66 (0.62)	*t*(409) = 33.03	<.001
Stimulant prescription: *N* (%)				
Current	0 (0%)	86 (36%)	*x*2(1) = 78.86	<.001
Lifetime	0 (0%)	96 (41%)	*x*2(1) = 92.47	<.001
Lifetime mood disorder: *N* (%)	4 (2%)	21 (9%)	*x*2(1) = 6.82	.009
Anxiety disorder: *N* (%)	12 (7%)	47 (20%)	*x*2(1) = 13.45	<.001
Oppositional defiant disorder: *N* (%)	1 (1%)	38 (16%)	*x*2(1) = 27.03	<.001

### Categorical ADHD status

**FIGURE 1:**
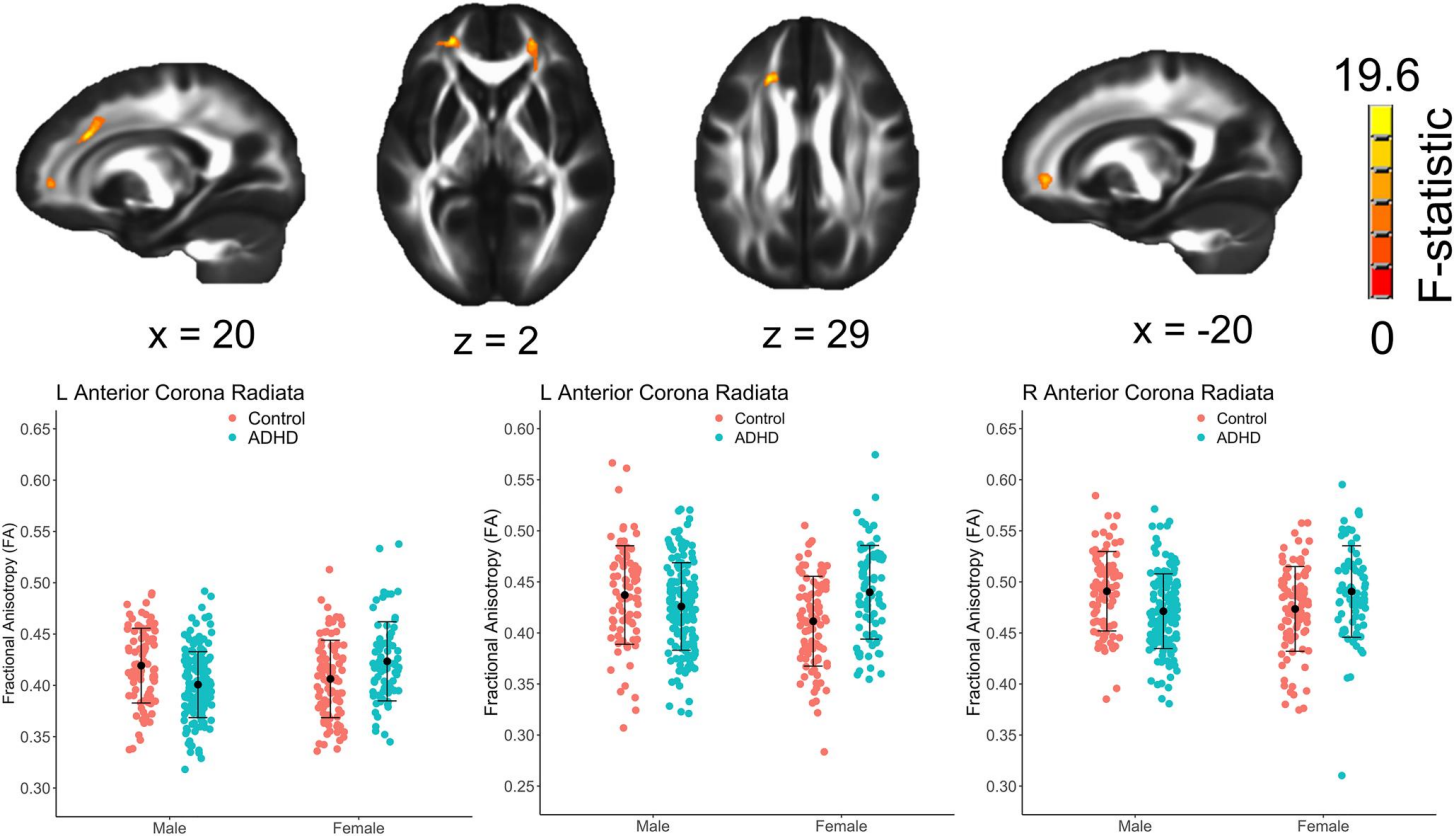
Sex differences in the association between categorical attention‐deficit/hyperactive disorder (ADHD) diagnosis and fractional anisotropy (FA). Significant clusters identified via voxel‐wise analyses (all voxel *p* < .01) looking at sex‐specific effects of ADHD diagnosis on FA. A sex‐by‐ADHD interaction was observed in three clusters. In the left and right anterior corona radiata (ACR) boys with ADHD had lower FA compared to boys without ADHD, but girls with ADHD had higher FA than girls without ADHD.

There were no regions where there was a significant main effect of ADHD status on FA when interactions with sex were ignored. However, three clusters, two in the left and one in the right ACR, showed a significant sex‐by‐ADHD diagnosis interaction (Table 2) where boys with ADHD demonstrated *lower* FA (all *b*s ≥ 0.012, *p*s < .05, all Cohen *d*s ≥ 0.246) and girls with ADHD had *higher* FA (all *b*s ≥ 0.017, *p*s < .01, all Cohen *d*s ≥ 0.394) compared to controls (Figure 1).

**TABLE 2:** Categorical and dimensional effects of attention‐deficit/hyperactive disorder (ADHD) on fractional anisotropy (FA)

Region	*X*	*Y*	*Z*	*F*‐stat	*η*2G	Voxels
Categorical diagnosis						
Sex‐by‐ADHD diagnosis interactions						
Right anterior corona radiata	−22	−45	3	14.98	0.04	665
Left anterior corona radiata	19	−24	30	18.15	0.04	430
Left anterior corona radiata	22	−46	2	19.57	0.05	366
Total symptom severity						
Main effect of ADHD symptom severity						
Left superior corona radiata	26	−8	24	14.53	0.03	649
Sex‐by‐ADHD symptom severity interactions						
Right anterior corona radiata	−20	−41	−1	18.21	0.04	846
Left anterior corona radiata	22	−46	2	20.41	0.05	587
Right cerebral peduncle	−23	22	−9	14.80	0.04	307
Inattention symptom severity						
Main effect of ADHD symptom severity						
Right superior longitudinal fasciculus	−37	33	30	19.47	0.05	612
Corpus callosum (body)	9	−16	23	15.15	0.04	392
Left ansa lenticularis	21	0	−6	17.22	0.04	344
Hyperactivity‐impulsivity symptom severity						
Main effect of ADHD symptom severity						
Left superior longitudinal fasciculus	26	11	39	14.00	0.03	362
Superior cerebellar peduncles	−3	37	−27	13.97	0.03	335
Right superior longitudinal fasciculus	−36	33	30	13.14	0.03	334
Sex‐by‐ADHD symptom severity interactions						
Left posterior corona radiata	32	31	29	15.73	0.04	454

*Note*: For all clusters identified during voxel‐wise analyses the location, *F*‐statistics, and Generalized Eta Squared (*η*2G), are provided for the voxel of peak effect.

### Dimensional ADHD symptom severity

**FIGURE 2:**
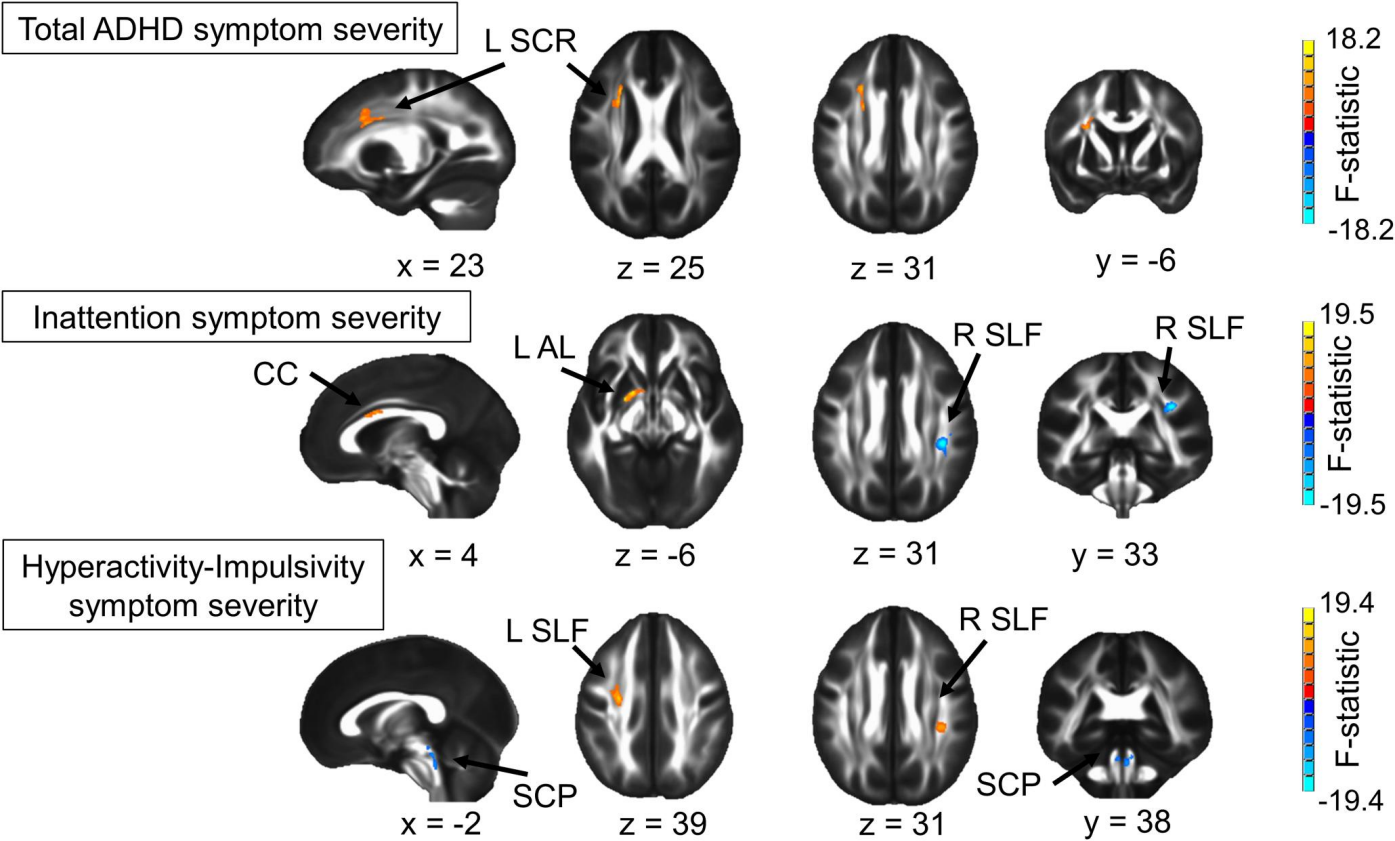
Dimensional associations between attention‐deficit/hyperactive disorder (ADHD) symptom severity and fractional anisotropy (FA). Significant clusters identified via voxel‐wise analyses (all voxel *p* < .01) looking at the main effect of dimensional associations between ADHD symptom severity and FA. Top: total ADHD symptom severity was positively associated (yellow/red colors) with FA in the superior corona radiata (SCR). Middle: inattention symptom severity was positively associated with FA in the corpus callosum (CC), and ansa lenticularis (AL), and negatively associated with FA in superior longitudinal fasciculus (SLF) (blue colors). Bottom: hyperactivity‐impulsivity symptom severity was positively associated with FA in the SLF and negatively associated with FA in the superior cerebellar peduncles (SCP).

**FIGURE 3:**
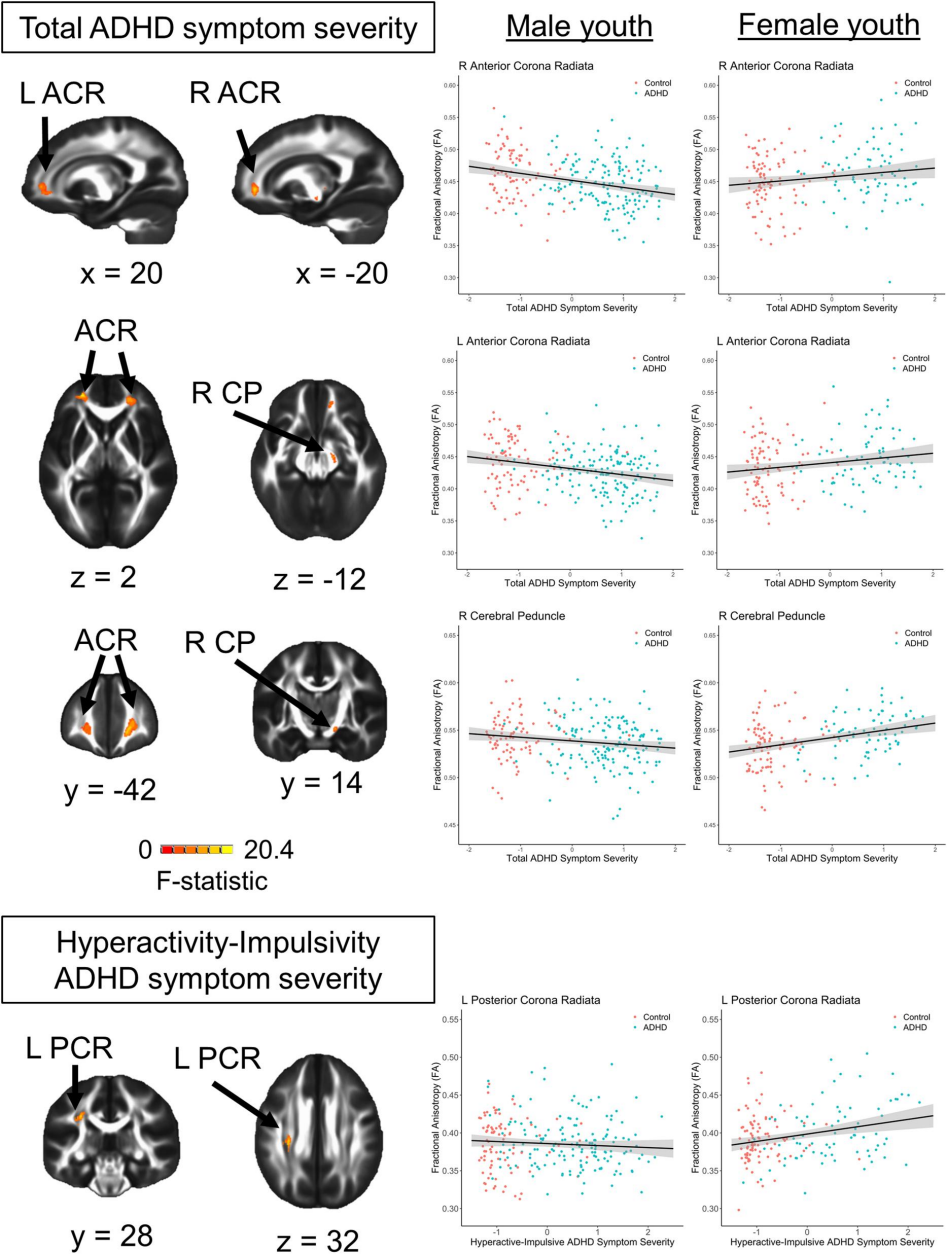
Sex differences in dimensional associations between attention‐deficit/hyperactive disorder (ADHD) symptom severity and fractional anisotropy (FA). Significant clusters identified via voxel‐wise analyses (all voxel *p* < .01) looking at sex‐specific dimensional associations between ADHD symptom severity and FA. Top: in the anterior corona radiata (ACR) and cerebral peduncle (CP), total ADHD symptom severity was associated with higher FA in girls but lower FA in boys. Bottom: in the posterior corona radiata (PCR), hyperactivity‐impulsivity symptom severity was associated with higher FA in girls, but was not associated with FA in boys.

Total ADHD symptom severity was associated with *higher* FA in the left superior corona radiata (SCR) (Figure 2). There was also a sex‐by‐total ADHD symptom severity interaction in three clusters: bilateral ACR (consistent with the diagnostic group findings) and right cerebral peduncle (CP). Here, total ADHD symptom severity was associated with *lower* FA in boys (all *b*s ≥ 0.004, *p*s < .01, *β*s ≥ 0.185) and *higher* FA in girls (all *b*s ≥ 0.007, *p*s < .05, *β*s ≥ 0.176) (Figure 3).

Inattention was associated with *higher* FA in the body and splenium of the corpus callosum (CC) and left ansa lenticularis (AL), and *lower* FA in the right SLF (Figure 2). There were no significant sex‐by‐inattention interaction effects.

Hyperactivity‐impulsivity was associated with *higher* FA in the bilateral SLF, and *lower* FA in the superior cerebellar peduncles (SCP) (Figure 2). There was a sex‐by‐hyperactivity‐impulsivity interaction in the left posterior corona radiata (PCR), such that symptom severity was associated with *higher* FA in girls (*b* = 0.019, *p* < .001, *β* = 0.524) but not boys (*p* > .05) (Figure 3).

### Sensitivity analyses

Sensitivity analyses confirmed all findings reported above remained statistically significant (all *p*s < .01) in post‐hoc models that included covariates for lifetime mood, anxiety, oppositional defiant disorders, current and lifetime stimulant medication prescription, signal‐to‐noise ratio, number of useable diffusion volumes, and familial relatedness in a random‐effects structure (see Supporting Information S1).

## DISCUSSION

This study confirms key associations with ADHD and alterations in white matter microstructure, in (to our knowledge) the largest case‐control sample to date. Further, it adds new information regarding symptom‐dimension specific associations and sex‐specific effects, which help to clarify prior contradictions in the literature.

Importantly, these findings provide strong support for previous reports of sex‐specific patterns of FA in ADHD, particularly in the prefrontal cortex (Jacobson et al., 2015; King et al., 2015). As hypothesized, girls with ADHD showed *higher* FA, while boys with ADHD showed *lower* FA (or no differences) compared to their peers in the bilateral anterior and left PCR and right CP. The neuromechanistic phenomena behind this sex‐specific effect is yet unclear; as FA may be related to alterations in fiber density, myelination, glial cells swelling, neuronal excitotoxicity, and neuronal branching at the gray‐white matter boundary (Hagmann et al., 2006; Jacobson et al., 2015; Lee et al., 2021). However, these results provide strong support for sex‐specific models of prefrontal cortical disruption in ADHD. While previous DTI studies in those with ADHD have attempted to control for sex‐specific effects, these studies have been highly unbalanced in male/female distribution. Covarying variables that differ at the population level is not recommended, and controlling for sex in these samples effectively co‐varies out female‐specific effects. Future studies may benefit from recruiting more balanced samples that are sufficiently powered to model sex‐by‐ADHD interaction effects.

A second major finding is the importance of dissociating ADHD symptom domains. Categorical ADHD‐control group comparisons yielded no significant main effects. Similarly, consideration of dimensional measures of total ADHD symptoms revealed a single cluster in the SCR, a region containing crossing fibers from several white matter tracts. The relative lack of other group‐level or total symptom score findings, despite a larger sample size than most prior studies, suggests that any population effects here are very small when distinct symptom domains are not considered.

When inattention and hyperactivity‐impulsivity domains were considered separately, meaningful unique associations emerged. Most notably, a differential relationship based on symptom domain was present in the SLF. Here, after controlling for overlap with hyperactivity‐impulsivity, inattention was associated with lower FA in the right hemisphere, whereas hyperactivity‐impulsivity, after controlling for overlap with inattention, was associated with higher FA bilaterally. Previous studies have found that compared to controls, those with ADHD have both greater (e.g., Peterson et al., 2011) and lesser (e.g., van Ewijk et al., 2014) FA in the SLF. Our findings may suggest these discrepancies are related to domain‐specific effects, and differences in symptom presentations across samples. This is consistent with prior independent studies that found that FA in this region is positively associated with hyperactivity‐impulsivity symptoms (Wu et al., 2017) and negatively associated with inattention symptoms (Chiang et al., 2015). The SLF may also be broken down into two or more sub‐bundles with unique anatomical connectivity patterns and associated function (Schurr et al., 2020). While impossible to confirm with the current data, differential domain‐specific effects between subregions of the SLF may be important to ADHD neurobiology and associated behavioral outcomes (Mazzetti et al., 2022).

Findings also highlight the involvement of the left AL, CC, and SCP in ADHD. The AL subserves cortico‐striatal‐thalamic circuitry (Kita, 2010), which plays a critical role in self‐regulation in many psychiatric conditions (Peters et al., 2016). Higher functional connectivity in these regions has been observed in children with ADHD and associated with impaired spatial working memory (Mills et al., 2012), and higher FA may reflect hyperactivation of this circuitry contributing to lapses in concentration and symptoms of inattention.

Previous studies report both positive (van Ewijk et al., 2014) and negative (Albaugh et al., 2019) associations between total ADHD symptom scores and FA in the CC. Our findings suggest greater FA in this region is associated with symptoms of inattention but not hyperactivity‐impulsivity. However, the CC carries cortical‐cortical fibers between many functionally distinct brain regions. Studies utilizing more precise measures of structural connectivity are necessary to fully disentangle whether FA in this region is differentially associated with ADHD symptom domains.

Finally the SCP carry fibers between the cerebellum and brainstem (Perrini et al., 2013). Lower FA in this region has been associated with motor impairments in autism spectrum disorder, possibly due to impairments in feedforward motor control or motor learning process (Hanaie et al., 2013); however, to our knowledge this is the first study to extend this negative association between FA and hyperactivity‐impulsivity to youth with ADHD.

While the present study has several strengths—ADHD cases were well‐characterized, the sample was recruited from the community, avoiding clinic referral sampling biases, and findings were not explained by medication use or history, comorbid conditions, or other confounds—several limitations should be noted. First, this is a convenience sample, not an epidemiological sample, so generalizability is unknown. Further, data here are cross‐sectional; it is unclear if FA alterations in association with ADHD were present prior to onset of symptoms, manifest over the course of the disorder, or persist later in life. Additionally, we chose to constrain our report to FA, as it is one of the most common metrics used to‐date, and our diffusion sequence (single‐shell sequence with 30 diffusion directions) limits our ability to carry out more advanced analytic techniques. However, tensor metrics are fairly limited in their interpretability and future studies should utilizing more advanced multi‐shell sequences, will be better equipped to handle issues of crossing fibers (e.g., Pines et al., 2020). Finally, the potential of differential patterns within subgroups of youth with ADHD, such as those with comorbid conditions (van Ewijk et al., 2016), were not examined directly. However, comorbid conditions did not account for the present results (see Supporting Information S1).

The issue of sample size also remains important. The size of ADHD groups in all but one (van Ewijk et al., 2014) of the exploratory whole‐brain studies of FA, cited above, range from 12 to 83 youth. Other large case‐control cohorts are available and will be informative but are smaller than the samples reported on here (e.g., Neuroimaging of the Children's Attention Project sub‐study; Silk et al., 2016). Meanwhile, the largest published population‐based study to‐date of FA (*n* = 1471) was an epidemiological sample that included a small proportion of children with ADHD (Albaugh et al., 2019). Other large, national cohorts (e.g., Adolescent Cognitive and Brain Development study) will provide important complementary information but similarly have overally low rates of ADHD (Cordova et al., 2022) and lack the detailed diagnostic characterization possible in case‐control designed studies. Thus, the present study had more power than most to‐date. Given recent work suggests that true population effect sizes for brain metrics in ADHD may be smaller than previously believed (Postema et al., 2021) and that larger confirmatory samples are needed (Marek et al., 2020), replication and extension to larger cohorts, as they become available, will be necessary.

Overall, two major conclusions emerge. First, while categorical findings were not present, more sensitive dimensional analyses using separate symptom domains showed unique FA correlates and, at times, opposite patterns of correlation, highlighting the multi‐componential nature of the disorder. Second, results strongly support sex‐specific effects in which girls with ADHD had higher FA, but boys with ADHD had lower FA, than typically‐developing peers of the same sex, particularly in the prefrontal cortex. These results underscore the importance of examining symptom domains and sex differences in studies of white matter in ADHD.

## AUTHOR CONTRIBUTIONS

**Scott A. Jones**: Conceptualization; Data curation; Formal analysis; Methodology; Visualization; Writing – original draft; Writing – review & editing. **Bonnie J. Nagel**: Conceptualization; Data curation; Funding acquisition; Methodology; Project administration; Writing – review & editing. **Joel T. Nigg**: Conceptualization; Data curation; Funding acquisition; Methodology; Project administration; Supervision; Writing – review & editing. **Sarah L. Karalunas**: Conceptualization; Data curation; Methodology; Project administration; Supervision; Writing – original draft; Writing – review & editing.

## CONFLICT OF INTEREST

Joel T. Nigg serves on the JCPP *Advances* Editorial Advisory Board. The remaining authors have declared that they have no competing or potential conflicts of interest.

## ETHICAL CONSIDERATIONS

All study procedures were approved by the Oregon Health & Science University Institutional Review Board. During recruitment, parents/legal guardians and children provided written consent and assent, respectively.

## Supporting information

Supporting Information S1

## Data Availability

All data will be uploaded and accessible via the National Institute of Mental Health's National Data Archive via collections associated with R37MH059105 (https://nda.nih.gov/edit_collection.html?id=3222) and R01MH115357‐01 (https://nda.nih.gov/edit_collection.html?id=2857).
