# Supplementary material for: Attention‐deficit/hyperactivity disorder and white matter microstructure: The importance of dimensional analyses and sex differences
Source: JCPP Adv. 2022 Nov 15;2(4):e12109. doi: 10.1002/jcv2.12109 (PMC9937645; doi:10.1002/jcv2.12109)
Supplement: Supplementary file 1 — Supporting Information S1 [file JCV2-2-e12109-s001.docx]

**Supporting information for: Attention-deficit/hyperactive disorder and white matter microstructure: the importance of dimensional analyses and sex differences**

Scott A. Jones^1^, Bonnie J. Nagel^1,2^, Joel T. Nigg^1,2^, Sarah L. Karalunas^3^

Table of Contents

[Sample selection 2](#_Toc118365114)

[ADHD diagnostic assignment 2](#_Toc118365115)

[Estimation of ADHD symptom scores 3](#_Toc118365116)

[The effect of image quality on fraction anisotropy 4](#_Toc118365117)

[The effect of volume censoring on fractional anisotropy 5](#_Toc118365118)

[Subject exclusion based on image quality 6](#_Toc118365119)

[Volume censoring and signal-to-noise ratio as a function of ADHD 6](#_Toc118365120)

[Generation of a study specific template 7](#_Toc118365121)

[Examination and removal of outliers in fractional anisotropy 8](#_Toc118365122)

[Voxel-wise results without modeling sex 8](#_Toc118365123)

[Categorical ADHD group differences 9](#_Toc118365124)

[Total ADHD symptom severity 9](#_Toc118365125)

[Inattention and hyperactivity-impulsivity dimensional severity 9](#_Toc118365126)

[Sensitivity analyses 10](#_Toc118365127)

[Lifetime mood disorder 10](#_Toc118365128)

[Anxiety disorder 10](#_Toc118365129)

[Oppositional defiant disorder (ODD) 11](#_Toc118365130)

[Current stimulant prescription 12](#_Toc118365131)

[Lifetime stimulant prescription 12](#_Toc118365132)

[Signal-to-noise ratio 13](#_Toc118365133)

[Number of remaining diffusion volumes after motion scrubbing 14](#_Toc118365134)

[Family relatedness 14](#_Toc118365135)

[References 16](#_Toc118365136)

[Table S1. Main effect of sex on fractional anisotropy 18](#_Toc118365137)

[Table S2. Association between ADHD symptoms and fractional anisotropy (without sex differences) 19](#_Toc118365138)

[Figure S1. The effect of image quality on fractional anisotropy 20](#_Toc118365139)

[Figure S2. White matter mask utilized for all voxel-wise analyses 21](#_Toc118365140)

[Figure S3. Sex differences in fractional anisotropy 22](#_Toc118365141)

# **Sample selection**

A total of 618 children were selected for the neuroimaging portion of an ongoing longitudinal examination of ADHD and adolescent development. During final sample selection for this manuscript, participants were excluded for the following reasons:

- 28 participants did not complete a full diffusion-weighted imaging and/or diffusion field map sequence during their MRI session.
- 89 participants completed one of several alternative, or modified, diffusion-weighted imaging sequences, all of which were sufficiently different from the one included in the final analyses.
- 33 participants were excluded as they did not meet criteria for ADHD or non-ADHD groups and fell into two sub categories: sub-threshold ADHD diagnosis (n = 15) or no ADHD diagnosis, but did not meet inclusion criterion to qualify as a control participant (n = 18).
- 57 participants were excluded during quality assessment due to scanner artifacts, excessive motion, or issues derived from imaging sequence application or image processing (detailed below).

These exclusions resulted in a final sample of 411 participants (234 with ADHD, and 177 without ADHD).

# **ADHD diagnostic assignment**

ADHD diagnoses in this non-referred sample were established in a multi-step, multi-method, multi-informant best estimate assessment process. Parents and teachers completed reliable and valid, standardized, nationally normed, widely accepted rating scales, including: the Conners' Rating Scales, 3^rd^ edition CRS-3 (Conners, 2003), Strengths and Difficulties Questionnaire long form including the impairment module (SDQ) (Goodman, 2001), and the ADHD Rating Scale for DSM-IV (ADHD-RS) (data collection began prior to DSM-5 publication) (DuPaul et al., 1998). Parents also completed semi-structured clinical interview administered by a trained Master’s-degree level clinician (Kiddie Schedule for Affective Disorders and Schizophrenia; K-SADS) (Puig-Antich and Ryan, 1986). Children completed a brief unstructured clinical interview, the three-subtest short form of the WISC-IV Vocabulary, Block Design, and Information (Wechsler, 2003, Sattler and Dumont, 2004) and the Word Reading subtest of the WIAT-II (Wechsler, 2002), to estimate IQ and academic progress. Written behavioral observations were provided. Final diagnoses were made by an experienced diagnostic team (board certified psychiatrist and licensed clinical psychologist) who considered all the preceding information but were blind to future DTI scan results. Each clinician independently determined diagnosis of ADHD and all other comorbid disorders. Disagreements were resolved via case discussion and cases in which the primary diagnoses could not be resolved were excluded. These expert diagnostic assignments achieved acceptable agreement for ADHD diagnosis (kappa > 0.88) and all other disorders with > 5% base rate in the same (kappa > 0.70).

# **Estimation of ADHD symptom scores**

Latent variable scores for ADHD symptom severity were computed as described previously (Nigg et al., 2020). The latent variable for overall ADHD symptom severity included hyperactivity-impulsivity and inattention raw scores from the CRS-R, ADHD-RS, and Strengths and Weaknesses of Attention-Deficit/Hyperactivity-symptoms and Normal-behaviors (SWAN) rating scale; the hyperactivity-inattention scale from the SDQ; and the hyperactivity-impulsivity and inattention symptom counts from the KSAD. For analyses focused on separate symptom dimensions, two separate latent variables were estimated: 1) inattention (indicators: inattention subscale raw scores from the CRS-R, ADHD-RS, and SWAN, as well as the KSAD inattention symptom count) and 2) hyperactivity-impulsivity (indicators: hyperactivity-impulsivity raw scores from the CRS-R, ADHD-RS, and SWAN; the SDQ hyperactivity-inattention scale raw score; and the KSAD hyperactivity-Impulsivity symptom count). As would be expected, both models fit the data well in the full sample (Nigg et al., 2020).

# **The effect of image quality on fraction anisotropy**

The effect of image quality on fractional anisotropy was examined in 98 participants. These participants were selected as they had at least one ‘Excellent’ (no motion-related artifacts) or ‘Good’ (20% or less of volumes with motion-related artifacts) quality run and at least one ‘Poor’ (>20% of volumes with motion-related artifacts) quality run. Diffusion weighted images were processed as described for the main analyses; however, no volumes were excluded for motion-related artifacts, and following motion and eddy-induced distortion correction, the concatenated runs were split and the diffusion tensor was estimated, and FA was calculated, for each run individually. These runs were then registered to the study-specific template and then to MNI space, and smoothed using a Gaussian kernel, as described in the main analyses.

A within-subjects comparison of runs that ‘passed’ quality assessment (Excellent/Good) to runs that ‘failed’ quality assessment (Poor) was carried out voxel-wise using a paired t-test in via AFNI’s 3dttest++. Similar to the primary analyses, a voxel threshold (p < 0.01) and cluster-forming (alpha < 0.05) FWE-correction was applied to the resulting z-statistic maps.

Similar to a previous between-subjects comparison using these quality assessment cutoffs (Roalf et al., 2016) we found four clusters (893 to 3,568 contiguous voxels) where FA was significantly greater in the runs that passed quality assessment compared to those that failed (Figure S1). These clusters were primarily located throughout the corpus callosum, left and right anterior corona radiata, right anterior limb of the internal capsule, and left posterior thalamic radiation. These results suggest that the inclusions of ‘Poor’ quality data will result in negatively biased FA measures in our sample.

# **The effect of volume censoring on fractional anisotropy**

The effect of volume censoring on fractional anisotropy was examined in 126 participants. These participants were selected because they had at least one Excellent (no motion-related artifacts) and one ‘Good’ (20% or less of volumes with motion-related artifacts) quality run. Data were processed exactly as described in the main analyses, including censoring of volumes with motion-related artifacts from ‘Good’ quality runs; however, runs were split prior to estimating the diffusion tensor, and fractional anisotropy was calculated for each run individually. Data were registered to MNI space and smoothed as described previously.

A within-subjects comparison of ‘Excellent’ quality runs and ‘Good’ quality runs was carried out voxel-wise using a paired t-test in via AFNI’s 3dttest++. Similar to the primary analyses, a voxel threshold (p < 0.01) and cluster-forming (alpha < 0.05) FWE-correction was applied to the resulting z-statistic maps. Unlike the previous supplementary analysis, we found no regions where ‘Excellent’ runs were significantly different than ‘Good’ quality runs that included motion censoring up to 20% (6 volumes). These results suggest that the inclusions of subjects in our sample with up with 20% of their diffusion directions censored from all runs likely has little impact on the overall estimation of fractional anisotropy. This 20% cutoff corresponds with the “Poor” data cutoff utilized by other labs previously (Roalf et al., 2016) and allows for the retention of significantly more subjects in our sample (~88%) compared to excluding subjects with *any* diffusion directions excluded (~67%).

# **Subject exclusion based on image quality**

As noted above, diffusion-weighted imaging quality assessment resulted in the exclusion of 4 participants due to field of view application errors, 7 participants due to scanner-related artifacts, and 43 participants due to excessive motion-related artifacts. Two additional participants were excluded due to failed convergence during the motion and eddy current-induced field distortion correction step (that could not be corrected with additional iterations), and one additional participant (with ADHD) was excluded due to failed registration to standard space. In total, 57 participants (51 with ADHD and 6 without ADHD) were excluded during quality assessment and image analyses. Youth with ADHD who were excluded from DWI analyses were younger than those included in DWI analyses [*t*(283) = 260, *p* < 0.01], but did not differ significantly in their inattention [*t*(283) = 0.15, *p* = 0.874], hyperactivity-impulsivity [*t*(283) = 0.05, *p* = 0.958], or total [*t*(283) = 0.13, *p* = 0.896] ADHD symptom severity.

# **Volume censoring and signal-to-noise ratio as a function of ADHD**

After the motion scrubbing procedure, those with ADHD had fewer remaining useable volumes than those without ADHD (b = -9.45, p < 0.001), controlling for effects of sex and age. Total symptom severity (b = -4.40, p < 0.001), inattention symptom severity (b = -4.49, p < 0.001), and hyperactivity-impulsivity symptom severity (b = -4.66, p < 0.001), were all also negatively associated with the number of remaining useable volumes, controlling for effects of sex and age. Similarly, in the cleaned data, those with ADHD had a lower signal-to-noise ratio, compared to those without ADHD (b = -0.114, p < 0.05), and total symptom severity (b = -0.053, p < 0.05), inattention symptom severity (b = -0.054, p < 0.05), and hyperactivity-impulsivity symptom severity (b = -0.044, p = 0.056) were negatively associated with signal-to-noise, controlling for age and sex.

# **Generation of a study specific template**

Previous studies suggest that use of a group-wise template for registration of DTI images (as opposed to pairwise registration to standard space), can improve reliability and reduce misalignment during registration (Schwarz et al., 2014, Keihaninejad et al., 2012). Given the computational demand of template creation in large samples, it has been suggested that as few as 10 images may be used to generate an unbiased group-wise template (Datta et al., 2015). Therefore, a study specific template was generated using FA maps from 100 pseudo-randomly selected subjects with minimal motion. This included 50 youth with ADHD and 50 without ADHD, as well as 50 male youth and 50 female youth (25 per diagnostic condition). Participants selected for template spanned the entire age range of the DWI dataset and were age matched between ADHD and sex sub-groups. ADHD symptoms severity (total, inattention, and hyperactivity-impulsivity) in subjects utilized for template creation did not differ from that of the rest of the sample (all p’s > 0.05). This template was generated using a single rigid registration followed by four iterations of non-linear registration (Schwarz et al., 2014).

# **Examination and removal of outliers in fractional anisotropy**

For all significant clusters identified in voxel-wise analyses, subjects with outlying values of fractional anisotropy were identified via visual inspection of scatter plots (similar to those in Figure 3 & 4). For analyses looking at the effects of ADHD diagnosis on FA (Model 1), and the association between total ADHD symptom severity and FA (Model 2), there was a cluster in the cerebellum where youth with ADHD had significantly lower FA compared to those without and total ADHD symptom severity was negatively associated with FA. However, in both analyses there were four subjects identified as outliers. These subjects were > 3 standard deviations below the group mean, and had average FA values in this region that were < 0.15. Upon further inspection of the raw data, these subjects had limited brain coverage in the inferior region of the cerebellum (due to suboptimal field of view application), which negatively impacted their FA values in this region. When these four subjects were removed and voxel-wise analyses were rerun, there were no longer significant clusters identified in the cerebellum.

# **Voxel-wise results without modeling sex**

In order to compare findings in our current sample to past literature, which were often underpowered to examine sex differences and often carried out analyses in samples that were heavily male-based, we conducted analyses similar to the main manuscript, only without modeling the effects of sex or sex-by-ADHD interactions. AFNI’s 3dttest++ was used to examine categorical differences in FA between those with and without ADHD and for dimensional analyses looking at associations between FA and total, inattention and hyperactivity-impulsivity ADHD symptom severity. All other procedures were identical to those described in the main manuscript. Voxel-wise models included:

(1) FA ~ ADHD diagnosis + age

(2) FA ~ total ADHD symptom severity + age

(3) FA ~ inattention symptom severity + hyperactivity-impulsivity symptom severity + age

## *Categorical ADHD group differences*

Similar to the primary analysis, there were no group differences in FA between adolescents with and without ADHD when not taking into account sex differences in the association between ADHD and FA.

## *Total ADHD symptom severity*

Similar to the primary analysis, total ADHD symptom severity was positively associated with FA in the left superior corona radiata in the full sample (Table S2).

## *Inattention and hyperactivity-impulsivity dimensional severity*

Similar to the primary analysis, inattention symptom severity was positively associated with FA in the body of the corpus callosum and negatively associated with FA in the right superior longitudinal fasciculus (Table S2). However, there was an additional region, in the right anterior corona radiata, where inattention symptom severity was negatively associated with FA, which did not hold up in the primary analysis when assessing sex differences. Importantly, the cluster identified in primary analysis in the left ansa lenticularis was not evident in these results, and only appears to emerge when sex differences in FA are taken into account.

Similar to the primary analysis, hyperactive-impulsive symptom severity was positively associated with FA in the right and left superior longitudinal fasciculus. Further, the cluster identified in the body of the corpus callosum only appears to emerge when sex differences in FA are take into account.

# **Sensitivity analyses**

## *Lifetime mood disorder*

When including a binary covariate for lifetime mood disorder, the association between total ADHD symptom severity and higher FA in the left superior corona radiata (b = 0.008, p < 0.001), the association between inattention and lower FA in the right superior longitudinal fasciculus (b = -0.017, p < 0.001) and higher FA in the body of the corpus callosum (b = 0.015, p < 0.001) and left ansa lenticularis (b = 0.015, p < 0.001), and the association between hyperactivity-impulsivity and lower FA in the superior cerebellar peduncles (b = -0.009, p < 0.001) and higher FA in the left (b = 0.014, p < 0.001) and right (b = 0.015, p < 0.001) superior longitudinal fasciculus, all remained significant.

Finally, the sex-by-ADHD diagnosis effect in the left (bs ≥ 0.035, ps < 0.001) and right (b = 0.036, p < 0.001) anterior corona radiata, the sex-by-total ADHD symptom severity interaction in the left (b = 0.016, p < 0.001) and right (b = 0.018, p < 0.001) anterior corona radiata, and right cerebral peduncle (b = 0.012, p < 0.001), and the sex-by-hyperactivity-impulsivity interaction in the left posterior corona radiata (b = 0.025, p < 0.001), all remained significant.

## *Anxiety disorder*

When including a binary covariate for anxiety disorder, the association between total ADHD symptom severity and higher FA in the left superior corona radiata (b = 0.007, p < 0.001), the association between inattention and lower FA in the right superior longitudinal fasciculus (b = -0.016, p < 0.001) and higher FA in the body of the corpus callosum (b = 0.016, p < 0.001) and left ansa lenticularis (b = 0.015, p < 0.001), and the association between hyperactivity-impulsivity and lower FA in the superior cerebellar peduncles (b = -0.009, p < 0.001) and higher FA in the left (b = 0.014, p < 0.001) and right (b = 0.015, p < 0.001) superior longitudinal fasciculus, all remained significant.

Finally, the sex-by-ADHD diagnosis effect in the left (bs ≥ 0.035, ps < 0.001) and right (b = 0.036, p < 0.001) anterior corona radiata, the sex-by-total ADHD symptom severity interaction in the left (b = 0.016, p < 0.001) and right (b = 0.018, p < 0.001) anterior corona radiata, and right cerebral peduncle (b = 0.012, p < 0.001), and the sex-by-hyperactivity-impulsivity interaction in the left posterior corona radiata (b = 0.025, p < 0.001), all remained significant.

## *Oppositional defiant disorder (ODD)*

When including a binary covariate for ODD, the association between total ADHD symptom severity and higher FA in the left superior corona radiata (b = 0.007, p < 0.001), the association between inattention and lower FA in the right superior longitudinal fasciculus (b = -0.017, p < 0.001) and higher FA in the body of the corpus callosum (b = 0.016, p < 0.001) and left ansa lenticularis (b = 0.015, p < 0.001), and the association between hyperactivity-impulsivity and lower FA in the superior cerebellar peduncles (b = -0.010, p < 0.001) and higher FA in the left (b = 0.013, p < 0.001) and right (b = 0.014, p < 0.001) superior longitudinal fasciculus, all remained significant.

Finally, the sex-by-ADHD diagnosis effect in the left (bs ≥ 0.035, ps < 0.001) and right (b = 0.037, p < 0.001) anterior corona radiata, the sex-by-total ADHD symptom severity interaction in the left (b = 0.017, p < 0.001) and right (b = 0.018, p < 0.001) anterior corona radiata, and right cerebral peduncle (b = 0.012, p < 0.001), and the sex-by-hyperactivity-impulsivity interaction in the left posterior corona radiata (b = 0.025, p < 0.001), all remained significant.

## *Current stimulant prescription*

When including a binary covariate for current stimulant prescription, the association between total ADHD symptom severity and higher FA in the left superior corona radiata (b = 0.008, p < 0.001), the association between inattention and lower FA in the right superior longitudinal fasciculus (b = -0.018, p < 0.001) and higher FA in the body of the corpus callosum (b = 0.012, p < 0.01) and left ansa lenticularis (b = 0.016, p < 0.001), and the association between hyperactivity-impulsivity and lower FA in the superior cerebellar peduncles (b = -0.009, p < 0.001) and higher FA in the left (b = 0.014, p < 0.001) and right (b = 0.015, p < 0.001) superior longitudinal fasciculus, all remained significant.

Finally, the sex-by-ADHD diagnosis effect in the left (bs ≥ 0.034, ps < 0.001) and right (b = 0.035, p < 0.001) anterior corona radiata, the sex-by-total ADHD symptom severity interaction in the left (b = 0.016, p < 0.001) and right (b = 0.017, p < 0.001) anterior corona radiata, and right cerebral peduncle (b = 0.012, p < 0.001), and the sex-by-hyperactivity-impulsivity interaction in the left posterior corona radiata (b = 0.024, p < 0.001), all remained significant.

## *Lifetime stimulant prescription*

When including a binary covariate for lifetime stimulant prescription, the association between total ADHD symptom severity and higher FA in the left superior corona radiata (b = 0.007, p < 0.001), the association between inattention and lower FA in the right superior longitudinal fasciculus (b = -0.018, p < 0.001) and higher FA in the body of the corpus callosum (b = 0.013, p < 0.01) and left ansa lenticularis (b = 0.015, p < 0.001), and the association between hyperactivity-impulsivity and lower FA in the superior cerebellar peduncles (b = -0.009, p < 0.001) and higher FA in the left (b = 0.014, p < 0.001) and right (b = 0.015, p < 0.001) superior longitudinal fasciculus, all remained significant.

Finally, the sex-by-ADHD diagnosis effect in the left (bs ≥ 0.034, ps < 0.001) and right (b = 0.036, p < 0.001) anterior corona radiata, the sex-by-total ADHD symptom severity interaction in the left (b = 0.016, p < 0.001) and right (b = 0.017, p < 0.001) anterior corona radiata, and right cerebral peduncle (b = 0.012, p < 0.001), and the sex-by-hyperactivity-impulsivity interaction in the left posterior corona radiata (b = 0.024, p < 0.001), all remained significant.

## *Signal-to-noise ratio*

When including signal-to-noise ratio as a covariate, the association between total ADHD symptom severity and higher FA in the left superior corona radiata (b = 0.007, p < 0.001), the association between inattention and lower FA in the right superior longitudinal fasciculus (b = -0.017, p < 0.001) and higher FA in the body of the corpus callosum (b = 0.015, p < 0.001) and left ansa lenticularis (b = 0.014, p < 0.001), and the association between hyperactivity-impulsivity and lower FA in the superior cerebellar peduncles (b = -0.009, p < 0.001) and higher FA in the left (b = 0.014, p < 0.001) and right (b = 0.015, p < 0.001) superior longitudinal fasciculus, all remained significant.

Finally, the sex-by-ADHD diagnosis effect in the left (bs ≥ 0.034, ps < 0.001) and right (b = 0.036, p < 0.001) anterior corona radiata, the sex-by-total ADHD symptom severity interaction in the left (b = 0.016, p < 0.001) and right (b = 0.017, p < 0.001) anterior corona radiata, and right cerebral peduncle (b = 0.012, p < 0.001), and the sex-by-hyperactivity-impulsivity interaction in the left posterior corona radiata (b = 0.025, p < 0.001), all remained significant.

## *Number of remaining diffusion volumes after motion scrubbing*

When including the number of remaining volumes after motion scrubbing as a covariate, the association between total ADHD symptom severity and higher FA in the left superior corona radiata (b = 0.007, p < 0.001), the association between inattention and lower FA in the right superior longitudinal fasciculus (b = -0.017, p < 0.001) and higher FA in the body of the corpus callosum (b = 0.015, p < 0.001) and left ansa lenticularis (b = 0.014, p < 0.001), and the association between hyperactivity-impulsivity and lower FA in the superior cerebellar peduncles (b = -0.009, p < 0.001) and higher FA in the left (b = 0.014, p < 0.001) and right (b = 0.015, p < 0.001) superior longitudinal fasciculus, all remained significant.

Finally, the sex-by-ADHD diagnosis effect in the left (bs ≥ 0.035, ps < 0.001) and right (b = 0.036, p < 0.001) anterior corona radiata, the sex-by-total ADHD symptom severity interaction in the left (b = 0.016, p < 0.001) and right (b = 0.018, p < 0.001) anterior corona radiata, and right cerebral peduncle (b = 0.012, p < 0.001), and the sex-by-hyperactivity-impulsivity interaction in the left posterior corona radiata (b = 0.024, p < 0.001), all remained significant.

## *Family relatedness*

To ensure the inclusion of sibling dyads and triads did not influence our results we remodeled all findings using mixed-effects regression (and included a random intercept term for each family):

(1) FA ~ diagnosis + sex + diagnosis x sex + age + (1|family id)

(2) FA ~ total ADHD symptoms + sex + total ADHD symptoms x sex + age + (1|family id)

(3) FA ~ inattention symptoms + hyperactivity-impulsivity symptoms + sex + inattention symptoms x sex + hyperactivity-impulsivity symptoms x sex + age + (1|family id)

In these analyses, the association between total ADHD symptom severity and higher FA in the left superior corona radiata (b = 0.008, p < 0.001), the association between inattention and lower FA in the right superior longitudinal fasciculus (b = -0.018, p < 0.001) and higher FA in the body of the corpus callosum (b = 0.015, p < 0.001) and left ansa lenticularis (b = 0.014, p < 0.001), and the association between hyperactivity-impulsivity and lower FA in the superior cerebellar peduncles (b = -0.008, p < 0.001) and higher FA in the left (b = 0.015, p < 0.001) and right (b = 0.016, p < 0.001) superior longitudinal fasciculus, all remained significant.

Finally, the sex-by-ADHD diagnosis effect in the left (bs ≥ 0.032, ps < 0.001) and right (b = 0.035, p < 0.001) anterior corona radiata, the sex-by-total ADHD symptom severity interaction in the left (b = 0.015, p < 0.001) and right (b = 0.017, p < 0.001) anterior corona radiata, and right cerebral peduncle (b = 0.012, p < 0.001), and the sex-by-hyperactivity-impulsivity interaction in the left posterior corona radiata (b = 0.023, p < 0.001), all remained significant.

# **References**

CONNERS, C. K. (2003). *Conners’ rating scales: Revised technical manual,* New York, NY: Multi-Health Systems.

DATTA, S., STAEWEN, T. D., COFIELD, S. S., CUTTER, G. R., LUBLIN, F. D., WOLINSKY, J. S., NARAYANA, P. A., AT HOUSTON, M. A. C. & GROUP, C. I. (2015). Regional gray matter atrophy in relapsing remitting multiple sclerosis: Baseline analysis of multi-center data. *Multiple sclerosis and related disorders,* 4**,** 124-136.

DUPAUL, G., POWER, T., ANASTOPOULOS, A. & REID, R. (1998). *ADHD Rating Scale—IV: Checklists, Norms, and Clinical Interpretation,* NY, NY: Guilford Press.

GOODMAN, R. (2001). Psychometric properties of the strengths and difficulties questionnaire. *Journal of the American Academy of Child & Adolescent Psychiatry,* 40**,** 1337-1345.

KEIHANINEJAD, S., RYAN, N. S., MALONE, I. B., MODAT, M., CASH, D., RIDGWAY, G. R., ZHANG, H., FOX, N. C. & OURSELIN, S. (2012). The Importance of Group-Wise Registration in Tract Based Spatial Statistics Study of Neurodegeneration: A Simulation Study in Alzheimer's Disease. *PLoS One,* 7**,** e45996.

NIGG, J. T., KARALUNAS, S. L., GUSTAFSSON, H. C., BHATT, P., RYABININ, P., MOONEY, M. A., FARAONE, S. V., FAIR, D. A. & WILMOT, B. (2020). Evaluating chronic emotional dysregulation and irritability in relation to ADHD and depression genetic risk in children with ADHD. *Journal of child psychology and psychiatry,* 61**,** 205-214.

PUIG-ANTICH, J. & RYAN, N. (1986). *Kiddie schedule for affective disorders and schizophrenia,* Pittsburgh, PA: Western Psychiatric Institute.

ROALF, D. R., QUARMLEY, M., ELLIOTT, M. A., SATTERTHWAITE, T. D., VANDEKAR, S. N., RUPAREL, K., GENNATAS, E. D., CALKINS, M. E., MOORE, T. M., HOPSON, R., PRABHAKARAN, K., JACKSON, C. T., VERMA, R., HAKONARSON, H., GUR, R. C. & GUR, R. E. (2016). The impact of quality assurance assessment on diffusion tensor imaging outcomes in a large-scale population-based cohort. *Neuroimage,* 125**,** 903-919.

SATTLER, J. & DUMONT, R. (2004). *Assessment of Children: WISC-IV and WPPSI-III Supplement,* San Diego: Jerome Sattler Publisher, Inc.

SCHWARZ, C. G., REID, R. I., GUNTER, J. L., SENJEM, M. L., PRZYBELSKI, S. A., ZUK, S. M., WHITWELL, J. L., VEMURI, P., JOSEPHS, K. A. & KANTARCI, K. (2014). Improved DTI registration allows voxel-based analysis that outperforms tract-based spatial statistics. *Neuroimage,* 94**,** 65-78.

WECHSLER, D. (2002). *Wechsler Individual Achievement Test, 2nd Ed (WIAT-II) Examiner’s Manual,* San Antonio: Harcourt Brace.

WECHSLER, D. (2003). *Wechsler Intelligence Scale for Children, 4th Ed (WISC-IV) Technical and Interpretive Manual,* San Antonio: Harcourt Brace.

# **Table S1. Main effect of sex on fractional anisotropy**

| region | X | Y | Z | F-stat | η2G | voxels |
| --- | --- | --- | --- | --- | --- | --- |
| *female > male* |  |  |  |  |  |  |
| R SCR | -27 | 21 | 39 | 38.84 | 0.09 | 3117 |
| L SCR | 33 | 31 | 32 | 30.27 | 0.07 | 2250 |
| R PTR | -39 | 50 | 10 | 18.89 | 0.04 | 1740 |
| L PTR | 33 | 57 | 9 | 23.63 | 0.05 | 1723 |
| thalamus | -4 | 1 | 4 | 31.14 | 0.07 | 1403 |
| L RLIC | 41 | 30 | 6 | 43.79 | 0.10 | 1200 |
| R EC | -32 | 3 | -9 | 56.99 | 0.12 | 1187 |
| CC (splenium) | 0 | 39 | 17 | 22.33 | 0.05 | 972 |
| R MCP | -14 | 46 | -29 | 40.58 | 0.09 | 679 |
| L EC | 32 | 9 | -11 | 46.56 | 0.10 | 550 |
| R AL | -18 | 5 | -12 | 40.81 | 0.09 | 513 |
| L MCP | 13 | 46 | -29 | 34.49 | 0.08 | 507 |
| L SLF | 34 | 12 | 23 | 26.54 | 0.06 | 329 |
| *male > female* |  |  |  |  |  |  |
| L PLIC | 11 | 3 | -7 | 54.61 | 0.12 | 2030 |
| R PLIC | -12 | 2 | -5 | 66.71 | 0.14 | 1630 |
| L MCP | 23 | 57 | -35 | 35.42 | 0.08 | 1340 |
| R MCP | -28 | 56 | -39 | 29.80 | 0.07 | 1265 |
| R PLIC | -3 | 25 | -4 | 29.70 | 0.07 | 504 |
| L Cingulum | 7 | -36 | 10 | 23.90 | 0.06 | 394 |
| R Postcentral-WM | -18 | 40 | 55 | 25.87 | 0.06 | 388 |
| CC (splenium) | 12 | 33 | 15 | 18.53 | 0.04 | 387 |
| L Postcentral-WM | 19 | 46 | 52 | 18.18 | 0.04 | 384 |
| L ACR | 16 | -29 | 23 | 16.68 | 0.04 | 333 |
| R STG-WM | -42 | 25 | -1 | 22.65 | 0.05 | 332 |
| L STG-WM | 45 | 38 | 5 | 22.18 | 0.05 | 330 |
| L PLIC | 30 | 18 | 17 | 15.08 | 0.04 | 326 |
| L SCP | 4 | 37 | -16 | 18.75 | 0.04 | 308 |
| R ACR | -16 | -19 | 31 | 12.14 | 0.03 | 301 |

Ansa Lenticularis (AL); Anterior Corona Radiata (ACR); Corpus Callosum (CC); External Capsule (EC); Left (L); Middle Cerebellar Peduncle (MCP); Posterior Limb of the Internal Capsule (PLIC); Posterior Thalamic Radiation (PTR); Retrolenticular part of the Internal Capsule (RLIC); Right (R); Superior Cerebellar Peduncle (SCP); Superior Corona Radiata (SCR); Superior Longitudinal Fasciculus (SLF); Superior Temporal Gyrus (STG); White Matter (WM). Average voxel-wise effect sizes are reported as generalized eta-squared (η^2^G).

# **Table S2. Association between ADHD symptoms and fractional anisotropy (without sex differences)**

| region | X | Y | Z | t-stat | η^2^G | voxels | |
| --- | --- | --- | --- | --- | --- | --- | --- |
| *total symptom severity* | | | | | | |  |
| L SCR | 24 | -16 | 35 | 2.96 | 0.02 | 353 | |
| *inattention symptom severity* | | | | | | |  |
| R SLF | -43 | 43 | 38 | -3.27 | 0.03 | 722 | |
| CC (Body) | 4 | -7 | 27 | 3.18 | 0.02 | 376 | |
| R ACR | -21 | -42 | 0 | -3.71 | 0.03 | 321 | |
| *hyperactivity-impulsivity symptom severity* | | | | | | |  |
| L SLF | 26 | 9 | 40 | 3.53 | 0.03 | 345 | |
| R SLF | -34 | 34 | 30 | 3.20 | 0.02 | 324 | |

Anterior Corona Radiata (ACR); Attention-deficit/hyperactive disorder (ADHD); Corpus Callosum (CC); Left (L); Right (R); Superior Corona Radiata (SCR); Superior Longitudinal Fasciculus (SLF). Average voxel-wise effect sizes are reported as generalized eta-squared (η^2^G).


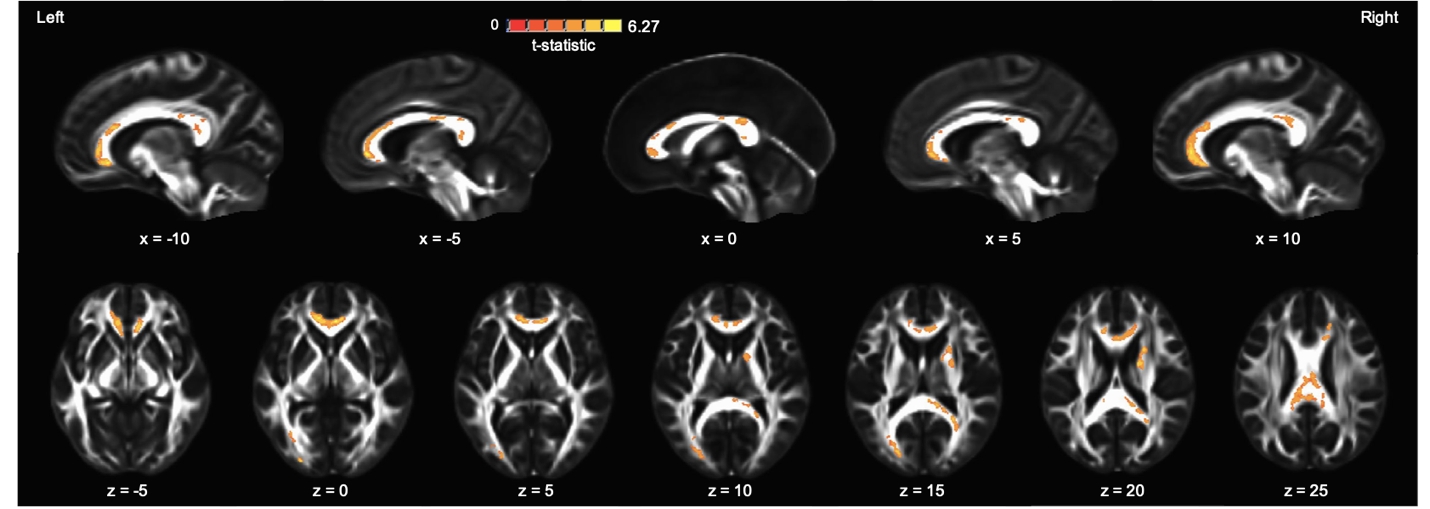


# **Figure S1. The effect of image quality on fractional anisotropy**

Brain regions where fractional anisotropy was greater in data that ‘passed’ (0-20% volumes containing motion-related artifacts) compared to ‘failed’ (>20% volumes containing motion-related artifacts) quality assessment.


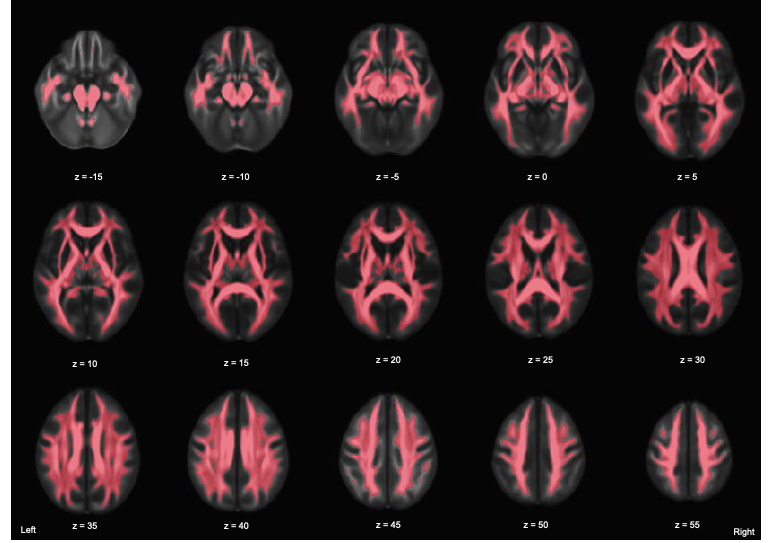


# **Figure S2. White matter mask utilized for all voxel-wise analyses**

Voxels in red represent all white matter voxels where mean FA across the entire sample was greater than 0.3.

**
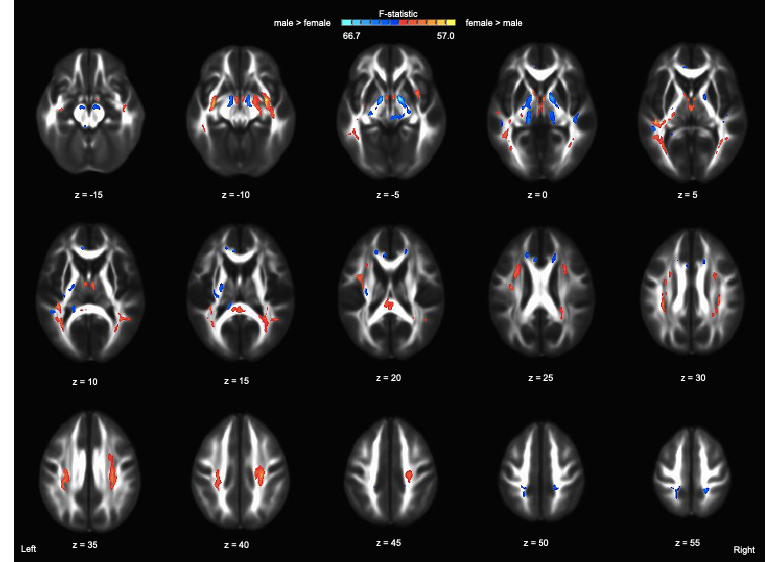
**

# **Figure S3. Sex differences in fractional anisotropy**

Main-effect of sex from the ADHD diagnosis-by-sex model (Model 1). There were widespread, bilateral regions where girls had greater FA (yellow-red) and lesser FA (blue) compared to boys.
